# Disparities in dietary intake and physical activity patterns across the urbanization divide in the Peruvian Andes

**DOI:** 10.1186/s12966-017-0545-4

**Published:** 2017-07-11

**Authors:** Morgan L. McCloskey, Carla E. Tarazona-Meza, Jessica C. Jones-Smith, Catherine H. Miele, Robert H. Gilman, Antonio Bernabe-Ortiz, J. Jaime Miranda, William Checkley

**Affiliations:** 1 Division of Pulmonary and Critical Care, School of Medicine, Johns Hopkins University, 1830 Monument St Room 555, Baltimore, MD 21205 USA; 2 Biomedical Research Unit, A.B. PRISMA, Lima, Peru; 3 Department of Health Services, University of Washington, Seattle, WA USA; 4 CRONICAS Center of Excellence in Chronic Diseases, Universidad Peruana Cayetano Heredia, Lima, Peru

**Keywords:** Nutrition transition, 24-h recall, Urbanization, Overweight, Low- and middle income countries, Chronic diseases

## Abstract

**Background:**

Diet and activity are thought to worsen with urbanization, thereby increasing risk of obesity and chronic diseases. A better understanding of dietary and activity patterns across the urbanization divide may help identify pathways, and therefore intervention targets, leading to the epidemic of overweight seen in low- and middle-income populations. Therefore, we sought to characterize diet and activity in a population-based study of urban and rural residents in Puno, Peru.

**Methods:**

We compared diet and activity in 1005 (503 urban, 502 rural) participants via a lifestyle questionnaire. We then recruited an age- and sex-stratified random sample of 50 (25 urban, 25 rural) participants to further characterize diet and activity. Among these participants, diet composition and macronutrient intake was assessed by three non-consecutive 24-h dietary recalls and physical activity was assessed using Omron JH-720itc pedometers.

**Results:**

Among 1005 participants, we found that urban residents consumed protein-rich foods, refined grains, sugary items, and fresh produce more frequently than rural residents. Among the 50 subsample participants, urban dwellers consumed more protein (47 vs. 39 g; *p* = 0.05), more carbohydrates (280 vs. 220 g; *p* = 0.03), more sugary foods (98 vs. 48 g, *p* = 0.02) and had greater dietary diversity (6.4 vs 5.8; *p* = 0.04). Rural subsample participants consumed more added salt (3.1 vs 1.7 g, *p* = 0.006) and tended to consume more vegetable oil. As estimated by pedometers, urban subsample participants burned fewer calories per day (191 vs 270 kcal, *p* = 0.03).

**Conclusions:**

Although urbanization is typically thought to increase consumption of fat, sugar and salt, our 24-h recall results were mixed and showed lower levels of obesity in rural Puno were not necessarily indicative of nutritionally-balanced diets. All subsample participants had relatively traditional lifestyles (low fat intake, limited consumption of processed foods and frequent walking) that may play a role in chronic disease outcomes in this region.

## Background

In recent years, the burden of disease in Peru has started to shift [1]. Generally described as the epidemiologic transition, deaths due to communicable diseases are falling, while deaths due to non-communicable disease are on the rise [1]. At the same time, Peru is undergoing a nutrition transition, which is broadly described as a shift in diet and physical activity patterns that results in an increasing prevalence of overweight and obesity [2–4]. A 2012 study assessed the nutrition transition in each Peruvian administrative region, based on anthropometric data [4]. Authors categorized the nutrition transition into three stages, with Stage 1 representing issues of chronic undernutrition, Stage 2 representing both undernutrition and overweight and Stage 3 representing primarily issues of overweight. Only the poorest region remained in Stage 1 of the nutrition transition [4]. A majority of regions (17) were in Stage 2, and seven regions were in Stage 3 [4]. Overall, it is estimated that overweight and obesity rates among adults in Peru currently range from 40 to 68% [3–5]. Increases in the number of individuals with excess weight is particularly concerning because obesity is associated with a substantially increased risk of diabetes, hypertension, coronary artery disease, and sleep apnea [6].

The nutrition transition is characterized by shifts in dietary intake, particularly increases in consumption of oils, caloric sweeteners, and animal products, which are thought to precede changes in disease burdens [2]. Between 1990 and 1999, the number of kilocalories available per capita in Peru increased from less than 2000 kcal per day to over 2500 kcal per day [7]. In the Andean region, there has been a significant decrease in consumption of vegetables, starchy roots, and fruit [7]. There has also been a shift from consumption of milk to greater consumption of soda and other sugar-sweetened beverages [5]. The nutrition transition also involves shifts in physical activity, typically toward more sedentary lifestyles [2]. Physical activity levels have decreased throughout the Latin American region and a national study on adults in all regions of Peru reported that 40% live a sedentary life [8]. Similarly, only 28% of Peruvian adults pursue vigorous physical activity [8].

A commonly cited driver of the nutrition transition and these lifestyle changes is urbanization, as individuals tend to shift from agrarian lifestyles to more sedentary jobs [9, 10]. Urbanization typically increases access to processed foods and street foods that are high in sugar, fat, and salt [9, 10]. The CRONICAS cohort, a longitudinal sample of over 3000 adults in four resource-poor settings in Peru, provides a platform to examine these patterns [11]. Rates of overweight and obesity in this cohort ranged from 76.6% in coastal, peri-urban Lima, 76.1% in urban Puno, 75.3% in coastal, semi-rural Tumbes and 48.1% in rural Puno. To better understand these discrepancies and determine if these lifestyle factors may be contributing to patterns of overweight/obesity, we conducted a pilot study on 50 participants in Puno, Peru to collect more detailed data regarding dietary intake and physical activity. We sought to characterize differences in dietary and physical activity patterns in urban and rural residents through the use of 24-h recalls and pedometers.

## Methods

### Study setting

This study was conducted in Puno, a city in southern Peru with approximately 150,000 inhabitants, at an elevation of 3825 m above sea level. The study population consisted of adults aged ≥35 years living in urban Puno city and surrounding rural communities. Urban participants commonly worked in commerce or education and lived in the city center, whereas rural participants were typically native subsistence farmers and lived in surrounding villages at varying distance from the city.

### Study design

Methods associated with the baseline questionnaire are described elsewhere [11]. A baseline lifestyle questionnaire provided information on general nutrition and physical activity among study participants. For the purpose of this study, we limited our analyses to participants in Puno. A total of 1005 participants answered the lifestyles questionnaire. Of these, we selected an age- and sex-stratified random sample of 25 urban and 25 rural participants for dietary intake (24-h recall) and physical activity (pedometer) measurements. Sample size calculations indicated that differences in macronutrient intakes and physical activity would have to be relatively large (331 kcal, 12 g fat, 12 g protein, 44 g carbohydrates and 3500 steps per day) to detect a significant difference with 50 participants, but dietary and physical activity information from the baseline questionnaire indicated there were substantial differences in consumption between urban and rural residents and we could reasonably expect a large effect size.

### Ethics approvals

The CRONICAS cohort study was approved by the internal review boards of the Johns Hopkins Bloomberg School of Public Health in Baltimore, MD, and Universidad Peruana Cayetano Heredia and A.B. PRISMA in Lima, Peru. This ancillary study was also approved by the same ethics boards as above and verbal informed consent was obtained and all participants received their sub-study results and generalized guidelines for improving their diet and physical activity habits.

### Dietary intake

Among the 1005 participants completing the lifestyle questionnaire, participants reported how frequently they consumed various food groups. From this, we calculated the average number of times per month participants consumed each food group. Food groups included in the questionnaire were representative of commonly eaten foods in this population as well as food groups that are commonly linked to chronic disease (food groups are listed in Table 1). Among the 50 subsample participants, diet was assessed with three, non-consecutive 24-h recalls, including one weekend day. All data collectors were trained together by a licensed nutritionist in Peru, who oversaw the data collection to ensure quality. Data collectors used one form to list all foods that participants consumed individually and another form to list all foods that participants consumed with others as part of a larger preparation. Participants identified portion sizes visually with a Peruvian standardized book that contained to-scale pictures of common food items, plate ware and corresponding gram weights [12]. If participants ate from a restaurant, ingredient weights were estimated using restaurant recipes. Supplement intake was not assessed.

**Table 1:** Characterization of body mass index, physical activity and average number of times various food groups were consumed per month among study participants in Puno, Peru

	Urban (*n* = 503)	Rural (*n* = 502)	*p*-value	Difference in multivariable analyses^b^	*p*-value
Mean age in years (SD)	55.3 (12.1)	55.5 (23.5)	0.76	-	-
% Males (n)	49.5 (249)	47.2 (237)	0.47	-	-
Mean wealth index (SD)	294 (7.95)	96.7 (3.38)	<0.001		
Average BMI in kg/m^2^ mean (SD)	27.9 (4.37)	25.2 (3.72)	<0.001		
BMI Categories^a^					
% underweight, (n)	0.59% (3)	1.6% (8)	<0.001	-	-
% normal weight, (n)	23.2 (117)	50.5(253)			
% overweight, (n)	49.7 (250)	37.1 (186)			
% obese, (n)	26.4 (133)	10.8 (54)			
Physical Activity					
Average days walking/week, mean (SD)	5.79 (1.96)	6.49 (1.51)	<0.001	0.75	<0.001
Average hours walking/day, mean (SD)	1.57 (2.08)	2.58 (2.11)	<0.001	0.94	<0.001
Food Groups					
Whole grains	14	12	0.25	0.84	0.47
Fruit	25	12	<0.001	−6.8	<0.001
Green vegetables	14	8.9	<0.001	−1.8	0.11
Cooked Vegetables	36	40	0.015	−0.47	0.77
Raw Vegetables	5.8	3.4	<0.001	−2.0	<0.001
Legume	4.6	6.5	0.001	1.7	0.02
Meat	15	16	0.28	2.1	0.22
Organ	4.2	3.4	0.06	−2.5	<0.001
Poultry	13	3.9	<0.001	−7.0	<0.001
Seafood	4.8	3.9	<0.001	−0.55	0.07
Eggs	12	10	0.06	−0.14	0.88
Potatoes	30.	48	<0.001	15	<0.001
Refined Grains	38	14	<0.001	−20	<0.001
Dairy	19	11	<0.001	−4.3	<0.001
Desserts	4.1	2.3	<0.001	−1.1	0.02
Ice Cream	3.1	2.9	0.51	0.14	0.72
Sugar	64	50	<0.001	−13	<0.001
Soda	7.2	4.6	<0.001	−2.4	0.002
Fruit Juice	9.6	3.1	<0.001	−3.9	<0.001
Fried Food	7.0	6.3	0.14	−0.59	0.325
Snacks	4.0	3.7	0.55	−1.0	0.15

^a^BMI categories defined by World Health Organization; underweight (BMI ≤ 18.5 kg/m2); normal weight (18.5 kg/m^2^ ≥ BMI ≤ 24.9 kg/m^2^); overweight (25 kg/m^2^ ≥ BMI ≤ 29.9 kg/m^2^); obese (BMI ≥ 30 kg/m^2^)

^b^Models were adjusted for age, sex, BMI and wealth index

Final gram weights of all food items consumed were calculated using an in-house developed software, which allowed for the calculation of waste factors for any foods not consumed in their entirety as well as individual consumption from larger preparations. All foods were codified according to the Peruvian food composition table, which was built into the software, and average macronutrient intakes were calculated for each participant. Due to limitations of the software and a Peruvian food composition table, micronutrient intakes were not included in the analysis. Any day in which participants consumed less than 400 cal was considered inaccurate and excluded from our analysis, resulting in the exclusion of 1 day from five urban participants and 1 day from one rural participant. Average macronutrient intake and diet composition were calculated for each individual based on mean consumption across the two (*n* = 6) or 3 days (*n* = 41) measured. Dietary diversity scores were calculated on a 0–9 scale based on the Food and Agriculture Organization of the United Nation’s guidelines for an individual-level Women’s Dietary Diversity Score [13]. Each food item was assigned to one of the following categories: starchy staples (white tubers and cereals); dark green leafy vegetables; vitamin A rich fruits and vegetables; other fruits and vegetables; organ meat; meat and fish; eggs; legumes, nuts and seeds; milk products. Additional categories were created to further assess diet: sugar (manually added); sugary foods (desserts, candy, soda, sugary drink mixes, etc); added salt and oil (vegetable oil, butter).

### Physical activity and anthropometrics

In the larger cohort, height and weight were assessed in triplicate using a locally-made stadiometer and a TF-300A body composition analyzer (TANITA Corporation, Japan), respectively, using standard techniques [11]. We re-assessed weight only in duplicate using the same balance in the sub-sample. In the lifestyle questionnaire, 1005 participants reported how much time they spent walking per week. Among the 50 subsample participants, physical activity was assessed with Omron HJ-720itc pedometers (Omron Healthcare, Inc., Bannockburn, USA). It has been shown that 3–5 days can sufficiently predict physical activity in adults, so participants were asked to wear the pedometers 5 days, including at least one weekend day [14–16]. Pedometers were programmed for each individual based on weight and average stride length. Pedometer data, including total steps, aerobic steps, calories burned, fat burned and distance walked, was downloaded into the Omron Health Management Software. The pedometer automatically calculated calories and fat burned based on a proprietary manufacturer formula. Pedometer data was discarded for any day in which participants wore the pedometer for less than 12 h. If this resulted in less than 3 days of usable data for a participant, these data were excluded from the analysis (*n* = 11). Average physical activity measures were calculated for each participant based on the number of days worn.

### Biostatistical methods

Two-sample t-tests with equal variances and multivariable linear regression, adjusted for age, sex, BMI and a wealth index [17] were used to compare baseline measures for the 1005 participants in Puno. Two-sample t-tests with equal variances were used to compare nutrient intakes and average grams consumed of each food group between subsample urban and rural participants. A Wilcoxon rank-sum test was used to compare the average grams consumed of each food group between all urban and rural participants. Due to the small sample size and to avoid parametric assumptions, a Kolmogorov-Smirnov test was used to compare pedometer measures between urban and rural participants. An alpha level of *p* < 0.05 was considered statistically significant. All statistical analyses were conducted in Stata 13 (StataCorp, College Station, Texas, USA).

## Results

### Participant characteristics

We selected a total of 4941 participants using a simple random sample from our community census, of which 2356 were not available for some reason and 276 did not meet the eligibility criteria. Of these, we contacted 2309 participants, and enrolled 1513 participants, of which 1005 completed all evaluations including lifestyle questionnaires and dietary data.

Among the 1005 participants with complete evaluations, 48.4% were male and average age was 55.4 years (Table 1). Urban participants had a higher average BMI when compared to rural participants (27.9 vs. 25.2 kg/m^2^; *p* < 0.001). Characteristics of the subsample were reflective of the entire Puno cohort. Specifically, the subsample was 52% male and the average age was 54.6 years (Table 2). On average, urban participants in the subsample had a slightly higher, albeit non-significant BMI and a higher wealth index (Table 2).

**Table 2:** General characteristics of the 50 urban and rural sub - study participants

	Urban (*n* = 25)	Rural (*n* = 25)	*p*-value
Mean age in years (SD)	57 (12)	51 (15)	0.10
Mean wealth index (SD)	311 (41)	124 (16)	<0.001
% male (n)	52 (13)	52 (13)	-
Average BMI in kg/m^2^, mean (SD)	26.9 (4.7)	25.2 (4.4)	0.18
BMI Categories^a^			0.30
% underweight, (n)	0 (0)	4 (1)	
% normal weight, (n)	44 (11)	48 (12)	
% overweight, (n)	32 (8)	40 (10)	
% obese, (n)	24 (6)	8 (2)	

^a^BMI categories defined by World Health Organization; underweight (BMI ≤ 18.5 kg/m2); normal weight (18.5 kg/m^2^ ≥ BMI ≤ 24.9 kg/m^2^); overweight (25 kg/m^2^ ≥ BMI ≤ 29.9 kg/m^2^); obese (BMI ≥ 30 kg/m^2^)

### Diet and physical activity in entire Puno cohort

After adjusting for age, sex, BMI and wealth index, urban residents consumed calorie-dense foods, such as refined grains, desserts, sugar, and soda more frequently (Table 1). Urban residents consumed a variety of protein-rich products, including poultry, organ meat and dairy, and fresh produce, including fruits and raw vegetables, more frequently than rural residents (Table 1). The only food groups consumed more frequently by rural residents were potatoes and legumes (Table 1). Rural residents walked more days per week (6.49 vs 5.79, *p* < 0.001) and spent more hours walking per day (2.58 vs 1.56, *p* < 0.001).

### Dietary intake in subsample

We obtained 24-h recall data for 47 participants. Urban participants consumed significantly more protein (47 g vs. 39 g, *p* = 0.048) and carbohydrates (280 g vs. 220 g, *p* = 0.033) than rural participants and tended to consume more calories (1600 kcal vs. 1300 kcal, *p* = 0.07). In terms of macronutrient composition, the diet of urban participants was 69.7% carbohydrates, 19.7% fat, and 11.9% protein; and, that of rural participants was 67% carbohydrates, 20.9% fat, and 11.7% protein. Macronutrient intakes did not appear differ between sexes or age categories (data not shown).

Urban participants had a higher average dietary diversity score of 6.4 compared to 5.8 among rural participants (*p* = 0.04) (Table 3). Food group categories utilized for the dietary diversity calculation were also used to characterize overall diet. Compared to rural participants, urban participants tended to consume a wider variety of fruits and protein-rich foods, including significantly more chicken (43 g vs. 14 g, *p* = 0.002) and dairy products (68 g vs. 25 g, *p* = 0.001) (Table 4). All participants consumed both cereal products and potatoes, although location played a significant role when it came to actual amounts consumed. Urban residents consumed significantly more cereal products (230 g vs. 140 g, *p* < 0.001), while rural participants consumed nearly triple the amount of potatoes (350 g vs. 130 g, *p* < 0.001) (Table 4). Rural participants consumed significantly more salt (3.1 g vs. 1.7 g, *p* = 0.006), although all 47 participants added salt to meals prepared at home (Table 4). Nearly all participants also added sugar to their food, with urban residents consuming more than double the amount of added sugar (20 g vs. 8.6 g, *p* = 0.11) and overall sugary products, such as candy, desserts, and sugary drink mixes (98 g vs. 46 g, *p* = 0.02) (Table 4).

**Table 3:** Average macronutrient intake per day from 3 non-consecutive 24-h recalls among urban and rural subsample participants

	Urban (*n* = 22)	Rural (*n* = 25)	*p*-value
Calories in kilocalories, mean (SD)	1600 (571)	1300 (445)	0.07
Protein in grams, mean (SD)	47 (15.4)	39 (14.1)	0.05
Fat in grams, mean (SD)	35 (18.9)	31 (21.9)	0.49
Carbohydrate in grams, mean (SD)	280 (111.7)	220 (63.3)	0.03
Dietary Diversity Score	6.4	5.8	0.04

**Table 4:** Proportion of participants consuming each food group and average grams consumed per day of each food group overall and among consumers from three non-consecutive 24-h recalls among urban and rural subsample participants

	Proportion Consuming			Average grams consumed overall			Average grams consumed among consumers		
Food Group	Urban (*n* = 22)	Rural (*n* = 25)	*p*-value	Urban (*n* = 22)	Rural (*n* = 25)	*p*-value	Urban (*n* = 22)	Rural (*n* = 25)	*p*-value
All meat	1.00	0.92	0.18	65	55	0.17	65	59	0.65
Chicken	0.91	0.36	0.001	43	14	0.002	47	39	0.61
Red meat	0.68	0.76	0.55	22	41	0.34	33	53	0.12
Fish	0.32	0.44	0.39	11	13	0.54	34	29	0.75
Dairy products	0.95	0.48	0.004	68	25	0.001	71	52	0.42
Eggs	0.55	0.36	0.20	7.5	9	0.29	14	26	0.18
Legumes	0.68	0.84	0.20	7.0	12	0.09	24	42	0.34
Cereals	1.00	1.00	-	230	140	<0.001	230	140	<0.001
White roots & tubers	1.00	1.00	-	130	350	<0.001	130	350	<0.001
Dark green vegetables	0.27	0.28	0.96	0.20	0.81	0.55	0.72	2.9	0.11
Vitamin A vegetables & tubers	0.95	0.96	0.92	43	42	0.96	45	44	0.88
Other vegetable	1.00	1.00	-	36	29	0.95	36	29	0.46
Vitamin A fruits	0.36	0.2	0.21	29	18	0.27	82	90.	0.88
Other fruits	1.00	1.00	-	160	120	0.79	160	120	0.49
Added salt	1.00	1.00	-	1.7	3.1	0.006	1.7	3.1	0.02
Added vegetable oil	0.82	0.88	0.55	6.9	14	0.15	8.5	16	0.08
Added sugar	0.91	0.92	0.89	20	8.6	0.11	22	9.4	0.12
All sugar & sugary foods	1.00	0.96	0.34	98	46	0.02	98	48	0.06

We had too small a sample to conduct multivariable analyses. However, in a two variable regression adjusting for wealth index, we found that the direction, magnitude and significance of our findings were unchanged.

### Physical activity in subsample

After accounting for non-compliance with study protocol, 39 participants remained for analysis. There were no significant differences between compliant and non-compliant participants with regard to site (45% vs 51% urban, *p* = 0.74), age (53.7 vs 58.1 years, *p* = 0.35), sex (54.5% vs 48.7% female, *p* = 0.74) or BMI (28.1 vs 25.4 kg/m^2^, *p* = 0.08). Among these participants, 20 wore their pedometers for 5 days, 10 wore their pedometers for 4 days and 9 wore their pedometers for 3 days. Rural participants burned more calories and walked longer distances than urban participants (Kolmogrov-Smirnov test *p* < 0.05). Although not statistically significant, rural participants tended to walk more steps per day and burn more fat (Table 5). Age was also a crucial factor influencing physical activity in this population, as all measures were significantly different between participants above and below age 60 years, with younger participants being more physically active (data not shown).

**Table 5:** Average pedometer results for urban and rural subsample participants

	Urban (*n* = 20)	Rural (*n* = 19)	*p*-value
Steps/day, mean (SD)	7641 (3459)	9519 (4586)	0.09
Aerobic steps/day, mean (SD)	1188 (1075)	1433 (1537)	0.38
Kcal burned/day, mean (SD)	191 (123)	270 (136)	0.03
Miles walked/day, mean (SD)	2.64 (1.51)	3.65 (1.95)	0.03
Grams fat burned/day, mean (SD)	10.71 (6.97)	15.22 (7.84)	0.08

## Discussion

In this high-altitude Andean population, location appeared to play an important role in diet and physical activity. In the entire Puno cohort, there were significant differences in the frequency of consumption of key food groups and frequency of physical activity between urban and rural populations. In our analysis of 24-h recalls from subsample participants, we found that urban participants consumed more grams of protein and carbohydrates than did rural participants, and tended to consume more calories. A majority of calories for all participants (67–70%) came from carbohydrates, indicating that participants consumed more than the recommended levels of carbohydrates relative to fat and protein, as this is above the Acceptable Macronutrient Distribution Range (AMDR) of 45–65% calories from carbohydrates [18]. Participants were on the lowest end of AMDR recommendations for fat and protein, with around 20% of calories derived from fat and around 12% of calories derived from protein.

In addition, other dietary studies have been completed in the Puno region [19–23]. Similar to our findings, many reported that rural residents received a substantial portion of their calories from potatoes or chuño, a stored and dehydrated potato [19–21]. In contrast, a study published in 1987 found that a majority of rural families did not have access to store-bought foods such as oatmeal, sugar, canned milk, and vegetable oil [19]. In our study, rural participants regularly consumed these food items, indicating the changes in the rural food landscape that have taken place in recent years. A systematic review on nutrition in the Central Andes region found that fat intakes in the reviewed reports were remarkably low, with multiple reports of percent energy from fat lower than 10% [24]. Although our data was not this extreme, with an average percent energy derived from fat around 20%, it is on the low end of macronutrient recommendations. Low fat intake may be a unique feature of Puno and other Andean populations compared to other regions of Peru. A 1990s study on nearly 800 individuals in urban and rural communities throughout Peru characterized diet based on 24-h recall and weighed record methods [25]. They found an average fat intake of 136 g, nearly quadruple the fat intake that was reported in our sample [25]. However, reported intakes of protein and carbohydrates were very similar to what was seen in our sample [25]. Lower fat intake could be an important factor in evaluating chronic disease risk in Puno adults compared to other individuals in the CRONICAS cohort.

In addition, through the 24-h recalls, we were able to characterize consumption of specific food groups and confirm dietary patterns seen in the entire Puno cohort. As seen in the baseline lifestyles questionnaire, urban residents consumed refined grains in the form of bread while rural residents consumed potatoes. A majority of rural residents received their protein from red meat products, including beef and alpaca. As seen in the baseline data, urban residents also consumed red meat, but tended to consume more chicken and dairy, so likely received protein from a wider variety of sources. Both subsample populations consumed a decent variety of vegetables in similar quantities. In nutrition transition literature, urbanization is often thought to increase consumption of sugar, salt, and fat [9, 10]. However, our results are mixed. As seen in the baseline data, urban residents did tend to consume more added sugar as well as other sugary products like cakes, sodas and chocolate. However, rural residents tended to consume more vegetable oil and added more salt to meals that they prepared. Overall, urban residents had a higher dietary diversity score, reflecting the greater variety of foods available in the urban environment.

In terms of physical activity, we also found that rural residents, regardless of age, burned more calories and walked longer distances each day. Although not statistically significant, rural residents also tended to complete more aerobic steps, indicating a greater intensity of activity, which could account for the greater caloric output in rural residents. Our findings are consistent with other studies on physical activity in Peru that report rural residents are more active as compared to urban residents. A study on migrants in Peru found that the prevalence of low physical activity was just 2.2% in rural residents, 32.2% in rural-urban migrants and 39.2% in urban residents [26].

An important strength of this study is that trained staff performed three, non-consecutive 24-h recalls, the gold standard for dietary intake assessment, on each participant. Food items were weighed whenever possible and participants used to-scale food pictures to most accurately identify what they had eaten. However, there is always the possibility of under-reporting with the 24-h recall technique, as individuals may not remember everything they consumed. Informal qualitative observations showed that individuals tended to underestimate the portion sizes of foods that they consumed. Authors of the systematic review on nutrition in the Andes reached similar conclusions about their data, supporting the idea that underreporting could be a crucial limitation to 24-h recall data in this region [25]. Another limitation of our study is that it was completed in the pre-harvest season. A study showed that mean energy intake of low socioeconomic status, rural Puno households was lower in the pre-harvest season, which could have contributed to limited dietary diversity and relatively low caloric intake reported by low-income rural residents in our study [23]. In terms of physical activity assessment, a key strength of this study was that participants wore the pedometer for a minimum of 3 days, so as to characterize average physical activity. We confirmed the feasibility of using pedometers in this population and there were no objections to pedometer use among participants. One limitation of pedometers in this setting is that they are not able to capture the full range of physical activities of the participants, particularly in rural areas where participants planted crops and carried extremely heavy loads. Due to the logistics of completing three non-consecutive 24-h recalls with each participant, the sample size was small and may not have been sufficiently powered to detect all differences. Despite large differences in weight status between urban and rural participants in the entire Puno cohort, there were no significant differences in BMI between the urban and rural sub-sample participants, which may have influenced the results. A final limitation is that, because this pilot sub-study was conducted only in Puno, generalizability is limited and findings are specific to high-altitude Andean populations.

This pilot study confirms and elaborates on the baseline diet and physical activity patterns identified in the Puno cohort. This study also demonstrates that lower levels of obesity in rural Puno are not necessarily indicative of nutritionally-balanced diets. Dietary diversity is positively associated with nutrient adequacy in both urban and rural populations [27, 28]. Because rural residents had significantly lower dietary diversity scores, it is likely that they are at higher risk of nutrient inadequacy than urban residents. In-field discussions with participants confirmed that rural residents had limited access to fresh produce and both the baseline and 24-h recall data showed that rural residents tended to consume a smaller variety and lower quantity of fruits and fresh vegetables. Additionally, rural residents consumed only 1300 kilocalories per day, well below recommendations for active Peruvian adults [29]. This data, in combination with findings that participants received nearly 70% of their daily calories from carbohydrates, indicates a need for further investigation into food security in rural areas and suggests that improving dietary diversity may be an important intervention target for the future. Future research on a larger number of individuals in this setting or across the entire CRONICAS cohort is necessary to further characterize diet and physical activity patterns and better identify intervention targets.

## Conclusions

Overall, both urban and rural subsample participants have relatively traditional lifestyles, with limited consumption of high-fat processed foods, moderate vegetable intake and relatively active lifestyles. Overall fat intakes were quite low and few participants reported consuming fast food or processed items. Both urban and rural adults under the age of 60 years were walking around 10,000 steps per day, meeting widespread guidelines for physical activity [14]. This could, in part, be due to the fact that urban Puno currently has a much lower degree of urbanization than other urban centers in Peru. Future efforts to encourage the maintenance of these traditional lifestyles in the face of increasing urbanization will be important. It is also worth considering that adults in this sample ranged from 37 to 80 years of age, an age group that is more likely to have maintained a traditional diet and lifestyle. In-field observations showed that children often consumed more sweets and processed products, so future research should evaluate dietary patterns among the younger generation.
